# ZIF-8-coated 3D-printed PCL/ion-doped BCP scaffolds for enhanced bone regeneration

**DOI:** 10.1371/journal.pone.0347048

**Published:** 2026-06-11

**Authors:** Vahid Khademi Ardakani, Niloufar Boroumand, Ghasem Dini, Mohsen Golabi, Maryam Abdollahi Asl

**Affiliations:** 1 Department of Biotechnology, Faculty of Biological Science and Technology, University of Isfahan, Isfahan, Iran; 2 Department of Nanotechnology, Faculty of Chemistry, University of Isfahan, Isfahan, Iran; Siksha O Anusandhan University Institute of Technical Education and Research, INDIA

## Abstract

Large bone defects require bioactive, mechanically robust scaffolds for regeneration. This study developed 3D-printed polycaprolactone/biphasic calcium phosphate (PCL/BCP) composite scaffolds (35−45 wt% ion-doped BCP with Sr^2+^, Mg^2+^, and Si^4+^) via fused deposition modeling (FDM), followed by in situ ZIF-8 coating. The optimal PCL + 40 wt.% ion-doped BCP formulation exhibited superior compressive strength (~30 MPa) and modulus (~ 0.4 GPa). Degradation in Phosphate-buffered saline (PBS) showed ~8% mass loss over 28 days with stable pH. Immersion in simulated body fluid (SBF) revealed rapid apatite formation and sustained release of Ca^2+^, P, Mg^2+^, Si^4+^, and Sr^2+^ ions. SEM confirmed uniform nanoscale ZIF-8 deposition, enriching surfaces with Zn^2+^. In vitro assays with MG-63 cells and hBMSCs demonstrated that ZIF-8-coated scaffolds significantly enhanced cell adhesion, proliferation (MTT), cytoskeletal organization (DAPI/phalloidin), and mineralization (Alizarin Red S) compared to uncoated controls (p < 0.05). The synergistic integration of ion-doped BCP, 3D printing, and ZIF-8 coating yields a bioactive, biodegradable platform with excellent osteogenic potential for advanced bone tissue engineering.

## 1. Introduction

Bone is a highly specialized mineralized tissue that plays a crucial role in providing mechanical support for the body and enabling locomotion [1,2]. However, its natural self-healing capacity is insufficient in severe cases such as trauma, large defects, or bone diseases like osteoporosis [3]. Current clinical treatments rely on autografts and allografts, which remain the gold standard but are limited by donor shortage, immune rejection, secondary trauma, and infection risk [4,5]. Therefore, the development of synthetic alternatives has become essential.

For repairing large segmental and load-bearing defects, 3D porous tissue engineering scaffolds are required to provide interconnected pores and sufficient mechanical integrity for bone regeneration [6]. An ideal scaffold should permit cell adhesion, vascularization, and nutrient diffusion [7]. The main objective of bone tissue engineering (BTE) is to design implantable materials that can be remodeled by host cells to restore bone tissue. The success of such scaffolds depends on the adaptability of their components, structure, physicochemical properties, and functions to natural bone tissue, including biocompatibility, bioactivity (osteoconductivity, osteoinductivity, and osseointegration), biodegradability, and appropriate mechanical strength. Various materials, including metals, bioceramics, polymers, and composites, have been explored for BTE [4,8]. Among them, calcium phosphate (CaP) biomaterials are widely used in BTE and 3D printing due to their osteoconductivity and bioresorbability, though they are brittle under tension and shear [9,10]. CaP-based ceramics such as hydroxyapatite (HA), β-tricalcium phosphate (β-TCP), and biphasic calcium phosphate (BCP, a mixture of HA and β-TCP) have gained attention for bone substitutes owing to their excellent biocompatibility [7]. Recent studies emphasize the benefits of nanoscale bioceramics, inspired by bone’s nanostructured composition—an organic matrix reinforced with inorganic CaP nanocrystals. Conventional synthetic microscale CaP ceramics differ significantly in bioactivity, degradability, and mechanical properties from nanoscale forms; therefore, fabricating nanoscale ceramics enhances biological performance [5].

Biopolymers such as polycaprolactone (PCL) are widely used due to their biocompatibility and FDA approval. While PCL offers good mechanical strength and processability, it is bioinert, hydrophobic, and produces acidic degradation products that may cause inflammation [10,11]. Its lack of biodegradability and osteoconductivity can be addressed by incorporating BCP nanoparticles (HA or β-TCP), acting as nucleation centers to promote apatite formation. Consequently, polymer/ceramic composites have been developed to combine the advantages of both materials [10]. In particular, PCL/β-TCP composites are highly attractive due to their biocompatibility, degradation profile, osteoconductivity, and compatibility with additive manufacturing techniques like fused deposition modeling (FDM) [10]. Previous studies have shown that PCL/CaP composite scaffolds enhance cellular and physical properties. Additional ions such as Si, Mg, and Sr can be incorporated to further improve performance [12–15]. For instance, Mg-doped β-TCP scaffolds promote cell viability and angiogenesis; Si-doped scaffolds enhance protein adsorption and apatite formation; and Zn^2+^, Co^2+^, and Sr^2+^ improve osteogenesis and vascularization [16].

Metal-organic frameworks (MOFs) have recently emerged as promising materials for BTE due to their high porosity, tunable structure, and large surface area [17]. MOF-based composites—combining MOFs with polymers, ceramics, or metals—exhibit improved mechanical strength, biocompatibility, and bioactivity, making them ideal candidates for advanced bone regeneration [17]. Among them, zeolitic imidazolate framework-8 (ZIF-8), consisting of Zn^2+^ and 2-methylimidazole, has received particular attention [18]. Zinc plays a vital biological role, and imidazole is part of amino acid side chains [19]. ZIF-8 thin films, synthesized via aqueous methods, show excellent biocompatibility, pH sensitivity, and mechanical stability. They can deliver therapeutic biomolecules, enhance scaffold surface properties, promote osteogenic differentiation, and release Zn^2+^ ions to stimulate bone regeneration [20–22]. Furthermore, ZIF-8 coatings can protect cells and biomolecules from external stressors such as heat, UV, and mechanical damage [23–25].

Additive manufacturing (3D printing) has revolutionized the fabrication of complex ceramic and composite scaffolds [26]. Since the 1980s, several 3D printing technologies, including FDM and selective laser sintering (SLS), have been developed [27]. Among them, extrusion-based bioprinting, where bioink is extruded through a nozzle to form layer-by-layer structures, is the most accessible and widely used technique for bone scaffold fabrication [28]. It enables the production of large scaffolds with well-defined pore architectures, offering an effective balance between porosity and mechanical strength [29]. Numerous in vivo studies have demonstrated the potential of 3D-printed HA and TCP scaffolds for craniofacial bone regeneration [30]. In recent years, there has been growing interest in biodegradable synthetic polymer/ceramic composites. The incorporation of 3D-printing techniques has created fresh opportunities for progress in bone regeneration and remodeling, offering novel approaches to heal bone defects and reconstruct functional tissue in patients [31].

This research focuses on the development of PCL/BCP nanocomposite scaffolds containing 35–45 wt.% BCP enriched with Sr, Si, and Mg ions. Among the investigated compositions, the scaffold incorporating 40 wt.% BCP was identified as the optimal formulation for further studies. This selection was driven by the objective of designing a scaffold suitable for human trabecular (cancellous) bone, which is predominantly present in joint regions, the epiphyses of long bones (e.g., femur and tibia), and the internal bone structure. Comparative evaluation of nanocomposite scaffolds containing 35, 40, and 45 wt.% BCP revealed that the 40 wt.% BCP scaffold exhibited mechanical properties most closely matching those of the target bone tissue. Consequently, scaffolds with this composition, enriched with Sr, Si, and Mg ions, were further investigated for their biodegradability and bioactivity. Subsequently, the scaffolds were coated with ZIF-8, and their biological performance was assessed in both coated and uncoated states. The results demonstrate that these nanocomposite scaffolds exhibit enhanced mechanical performance, improved bioactivity, and controlled degradation behavior, addressing key challenges associated with the regeneration of load-bearing bone defects. Nevertheless, further in vivo studies and mechanical evaluations under physiological conditions are necessary to validate their clinical applicability.

## 2. Materials and methods

### 2.1. Materials

PCL (Mw = 80 kDa), zinc nitrate hexahydrate (Zn(NO_3_)_2_·6H_2_O), 2-methylimidazole (2-MIM), dopamine (DA), polyethylenimine (PEI), and tris(hydroxymethyl)aminomethane (Tris) buffer were purchased from Sigma-Aldrich (USA). Chloroform and dimethyl sulfoxide (DMSO) (Sigma-Aldrich, USA) were used as solvents. Phosphate-buffered saline (PBS) and simulated body fluid (SBF) were supplied by Ceram Razi Co. (Iran). For cell culture, DMEM-F12 medium (HyClone, GE Healthcare, USA) was supplemented with 10% fetal bovine serum (FBS) and 1% penicillin–streptomycin (Gibco, USA). The MG-63 human osteosarcoma and human bone marrow-derived mesenchymal stem cell (hBMSC) lines were obtained from the National Cell Bank of Iran (Pasteur Institute, Tehran, Iran). Additional reagents included MTT (Sigma-Aldrich, USA), Alizarin Red S, paraformaldehyde, Triton X-100, and acetic acid (all from Merck, Germany), as well as FITC–phalloidin and DAPI (Sigma-Aldrich, USA). Unless otherwise stated, all chemicals were used as received without further purification.

### 2.2. Fabrication of 3D-Printed PCL/BCP Scaffolds

#### 2.2.1. Synthesis of Ion-Doped BCP Powder.

BCP powder doped with Sr, Mg, and Si ions was synthesized according to previous works [32,33] via the co-precipitation method using calcium nitrate tetrahydrate, diammonium hydrogen phosphate, strontium nitrate, magnesium nitrate hexahydrate, and tetraethyl orthosilicate (TEOS) as precursors. Briefly, Ca, Mg, and Sr salts were dissolved in distilled water and added dropwise to a solution containing TEOS and diammonium hydrogen phosphate, while maintaining a Ca/P molar ratio of 1.6 and adjusting the pH to 10 using ammonia. The mixture was stirred at 90 °C for 2 h, dried at 50 °C, calcined at 650 °C for 2 h (heating rate: 5 °C/min), then ground and sieved (400 mesh) for subsequent characterization and scaffold fabrication.

#### 2.2.2. Fabrication of 3D-printed PCL/BCP composite scaffolds.

Composite scaffolds were produced by mixing ion-doped BCP powders (35, 40, and 45 wt.%) with a PCL matrix. PCL was first dissolved in chloroform and stirred for 1 h, while BCP nanopowders were separately dispersed in chloroform, stirred for 4 h, and sonicated for 30 min. The BCP suspension was then combined with the PCL solution and mixed on a magnetic stirrer at room temperature until the solvent fully evaporated (about 8 hours). The resulting blend was dried at 40 °C for 1 h to obtain uniform solid composites ready for printing. These composites were cut into small pieces and loaded into the syringe of a 3D printer (3DPL, Iran) for FDM extrusion. Porous cubic scaffolds were designed in Simplify3D, using a 500 μm stainless-steel nozzle. Optimized parameters included a printing speed of 90 mm/min, a melt temperature of 130 °C, and an extrusion pressure of 3.5 bar. After fabrication, the scaffolds were vacuum-dried to eliminate any remaining solvent and ensure structural integrity.

#### 2.2.3. Evaluation of mechanical properties.

Scaffold samples with three different BCP weight ratios were prepared for compressive testing using a 2T SANTAM universal testing machine. Five samples from each scaffold (dimensions: approximately 10 × 10 × 5 mm³) were measured for length, width, and height before testing. The samples were positioned such that the compressive load was applied perpendicular to the printing layers (build direction) to evaluate the out-of-plane mechanical performance. Compressive load was applied at a crosshead speed of 0.2 mm/s until 70% strain was achieved, at which point the test was terminated. Mechanical data analysis revealed that scaffolds containing 40 wt.% BCP nanoparticles and 60 wt.% PCL exhibited the highest compressive strength and the most favorable mechanical performance. Therefore, this composition was selected for the fabrication of PCL/BCP composite scaffold samples for subsequent investigations.

#### 2.2.4. Degradation evaluation.

The degradability of the scaffolds was evaluated according to a previously reported protocol [29]. Pre-weighed 3D-printed samples (n = 3) were placed in 10 mL of PBS at ~ 37 °C for selected time points (1, 3, 5, 7, 14, 21, and 28 days). At each interval, the samples were removed, dried in a vacuum oven at 40 °C for 48 h, and weighed again. Weight loss was determined by comparing the initial and final masses. The pH of the PBS medium was also recorded at each time point to track any fluctuations during degradation.

#### 2.2.5. Bioactivity evaluation.

As described in a previous publication [34], scaffold bioactivity was evaluated by immersing sterilized samples (n = 3) in 10 mL of SBF at ~ 37 °C for 1, 3, 5, 7, 14, 21, and 28 days. At each time point, SBF pH was measured to monitor bioactivity progression. The concentrations of released Ca, P, Mg, Si, and Sr ions in the SBF were quantified using inductively coupled plasma optical emission spectrometry (ICP-OES, Analytik Jena PQ 9000). Apatite formation on the scaffold surfaces was examined using scanning electron microscopy (SEM, Philips XL30) after 14 and 28 days of immersion in SBF.

### 2.3. Surface modification and In situ ZIF-8 coating of scaffolds

In this approach, the scaffold surface was first modified with a dopamine/polyethylenimine (DA/PEI) layer to provide an adhesive substrate for ZIF-8 growth, followed by in situ ZIF-8 synthesis. To enhance surface functionality and bioactivity, a hybrid DA/PEI coating was deposited via self-polymerization. A 10 mM Tris–HCl buffer (pH 8.5) was prepared, into which dopamine hydrochloride (2 mg/mL) and branched polyethyleneimine (PEI, 2 mg/mL, Mw ≈ 25 kDa) were dissolved under gentle stirring. Pre-cleaned scaffolds were immersed in the fresh DA/PEI solution and incubated in a shaker (Rotomix Behdad) at 50 rpm for 8 h to facilitate in situ oxidative polymerization of dopamine and its interaction with PEI through Schiff-base and Michael addition reactions. Coated scaffolds were thoroughly rinsed with distilled water to remove unbound residues and vacuum-dried at 37 °C overnight. The resulting DA/PEI layer provided abundant amine and catechol groups, improving surface hydrophilicity and creating active sites for subsequent biomineralization and functionalization.

ZIF-8 nanoparticles were then synthesized in situ on the DA/PEI-coated scaffolds using a mild aqueous method. Zinc nitrate hexahydrate and 2-methylimidazole were separately dissolved in double-distilled water. Scaffolds were immersed in the zinc precursor solution for 1 min to allow Zn² ⁺ adsorption, after which the 2-methylimidazole solution was added. The appearance of a milky-white suspension confirmed ZIF-8 nucleation. Samples were incubated at room temperature for 2.5 h without agitation to ensure uniform crystallization, then washed with distilled water and vacuum-dried at 37 °C.

Scaffolds were divided into two groups for biological evaluation: (1) optimized scaffolds without ZIF-8 (Scaffold 1) and (2) optimized scaffolds functionalized with ZIF-8 (Scaffold 2). Surface morphology after ZIF-8 deposition was characterized by SEM.

### 2.4. Cell experiments

#### 2.4.1. Cell viability assay.

The cytocompatibility of the scaffolds was evaluated using MG-63 cells via the MTT assay, following the protocol described in [29]. Briefly, sterilized scaffolds (~5 × 5 × 3 mm³; n = 3) were seeded with 1 × 10⁴ cells/mL and cultured in DMEM-F12 supplemented with 10% (v/v) FBS and 1% (v/v) penicillin–streptomycin at 37 °C in a 5% CO₂ atmosphere for 1, 3, and 5 days. Cells cultured without scaffolds served as the control. At each time point, the medium was replaced with 400 µL of serum-free medium and 40 µL of MTT solution (5 mg/mL in PBS), followed by incubation in the dark at 37 °C for 4 h. Formazan crystals were dissolved in DMSO, and absorbance was measured at 490 nm using a spectrophotometer to quantify mitochondrial activity. In addition, scaffold-only blank samples (scaffolds without cells) were processed under identical conditions to evaluate possible interference of the scaffold materials or degradation products with the MTT signal. The absorbance of these blanks was subtracted from the corresponding experimental readings for background correction. To minimize potential pH-related effects on the assay, the culture medium was refreshed regularly during the incubation period to maintain physiological pH.

#### 2.4.2. Fluorescence staining.

Sterilized scaffolds were seeded with MG-63 cells (1 × 10⁴ cells/well) and incubated for 3 days at 37 °C in 5% CO₂. Samples were washed with PBS, fixed in 4% paraformaldehyde, permeabilized with 0.25% Triton X-100, and stained with FITC–phalloidin (for cytoskeleton) and DAPI (for nuclei). Imaging was performed using an Olympus BX51 fluorescence microscope. Fluorescence images were then quantified using ImageJ software. For the DAPI channel, nuclei were segmented via automatic thresholding and watershed algorithm after background subtraction; cell density was calculated as nuclei count divided by scaffold area in the field of view. For the FITC-phalloidin channel, actin structures were segmented based on intensity threshold, followed by particle analysis to measure count, density, area, and size. At least 5–10 random fields per sample were analyzed (n = 3 scaffolds per group). Statistical comparisons used the unpaired Student’s t-test.

#### 2.4.3. Alizarin Red S staining.

Alizarin Red staining was used to qualitatively and quantitatively assess calcium deposition. Human bone marrow-derived mesenchymal stem cells (hBMSCs) were seeded at 1 × 10⁴ cells/mL on the scaffolds and cultured in osteogenic differentiation medium for 14 days. Cells were then fixed with 70% ethanol for 1 h and rinsed thoroughly with distilled water. Samples were incubated in 0.2% Alizarin Red solution (pH 4.2) at room temperature for 1 h. After washing with distilled water to remove unbound dye, scaffolds were air-dried at 30 °C for 5 min and examined under an optical microscope at randomly selected regions. For quantitative analysis, bound dye was eluted by immersing stained scaffolds in a solution containing 20% methanol and 10% acetic acid for 15 min. After complete dye dissolution, absorbance was measured at 405 nm using a microplate reader to quantify calcium deposition. Data are presented as mean ± standard deviation (SD). Comparisons between two groups were performed using an unpaired Student’s t-test.

### 2.5. Statistical analysis

All experiments were performed with three samples per group (n = 3), unless otherwise stated. For the MTT assay, data were analyzed using two-way analysis of variance (ANOVA), followed by Tukey’s post hoc test. Statistical analysis was conducted with GraphPad Prism software, and results are expressed as mean ± SD. A p-value < 0.05 was considered statistically significant.

## 3. Results and discussion

### 3.1. Mechanical properties

**Fig 1:**
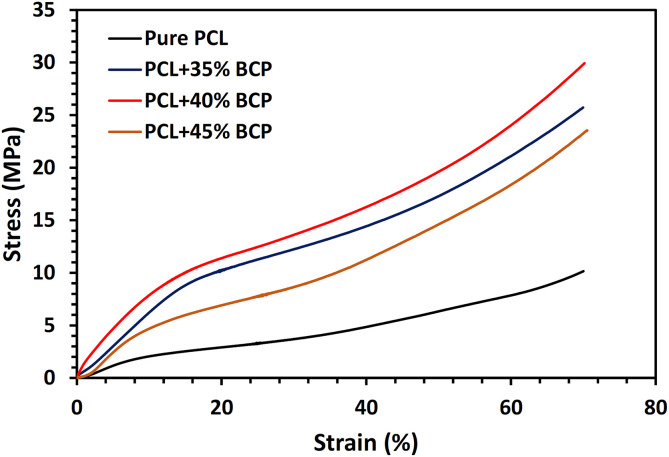
Compressive strain-stress curves of the fabricated scaffolds in this study.

Compression tests were conducted to determine how varying ion-doped BCP content influences the scaffolds’ compressive properties. The measured parameters—including compressive strength, yield strength, and modulus—are presented in Table 1. Corresponding stress–strain curves for pure PCL and composite scaffolds are displayed in Fig 1. All composite scaffolds exhibited similar stress–strain behavior. Pure PCL scaffolds displayed the lowest compressive modulus (0.18 MPa), consistent with the inherently soft and ductile nature of PCL. Incorporation of BCP nanoparticles significantly enhanced mechanical performance. The compressive modulus increased from 0.18 MPa for pure PCL to 0.48 MPa for the scaffold with 45 wt.% BCP. The PCL/40 wt.% BCP composition demonstrated the highest compressive strength (29.7 MPa), an optimal compressive modulus, and a favorable balance between stiffness and flexibility. Scaffolds with 35 and 40 wt.% BCP exhibited yield strengths of 7.8 MPa and 8.9 MPa, respectively—values suitable for BTE applications. In contrast, increasing BCP to 45 wt.% led to a reduction in yield strength (4.5 MPa) and compressive strength (23.4 MPa), likely due to nanoparticle agglomeration and structural inhomogeneity. Among all compositions, the PCL/40 wt.% BCP scaffold exhibited the most balanced mechanical profile and was therefore selected as the optimal formulation for subsequent analyses. Although the mechanical properties were evaluated in the initial state, assessing the evolution of mechanical performance during degradation and PBS immersion would provide a more comprehensive understanding of long-term scaffold stability and will be addressed in future studies.

**Table 1:** Mechanical properties of different scaffolds fabricated in this study.

Composition	Compressive modulus (GPa)	Yield strength (MPa)	Compressive strength (MPa)
Pure PCL	0.18 ± 0.03	1.8 ± 0.2	10.1 ± 0.5
PCL + 35 wt.% BCP	0.35 ± 0.04	7.8 ± 0.2	25.6 ± 0.4
PCL + 40 wt.% BCP	0.41 ± 0.03	8.9 ± 0.4	29.7 ± 0.3
PCL + 45 wt.% BCP	0.48 ± 0.05	4.5 ± 0.3	23.4 ± 0.5

### 3.2. Degradability

**Fig 2:**
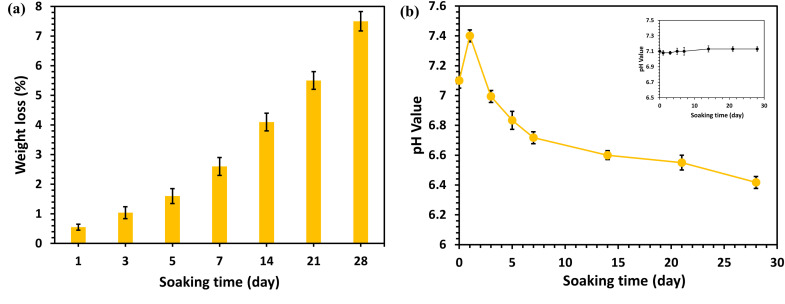
Degradation behavior of PCL/40 wt.% ion-doped BCP scaffolds during immersion in PBS. (a) Mass variation of PCL/40 wt.% ion-doped BCP scaffolds during immersion in PBS, and (b) pH changes of the PBS solution during 28 days of immersion. The inset in panel (b) shows the pH variation of PBS control without scaffolds over the same incubation period.

Fig 2 illustrates the degradation behavior of 3D-printed PCL/40 wt.% ion-doped BCP scaffolds in PBS over 28 days. Fig 2a shows the mass loss of the scaffolds, while Fig 2b presents the corresponding pH changes in the immersion medium. As shown in Fig 2a, scaffold mass decreased gradually, reaching ~8% weight loss by day 28. This reduction is attributed to the controlled release of BCP nanoparticles. Such progressive degradation is ideal for tissue-engineered scaffolds, as it maintains structural support while allowing cellular infiltration and gradual replacement with newly formed bone. Fig 2b reveals a steady decline in pH from 7.4 (day 1) to 6.4 (day 28). This acidification likely results from the dissolution of β-TCP within BCP, releasing Ca²⁺ and PO₄³⁻ ions and disrupting ionic equilibrium. Additionally, surface reprecipitation of apatite or phosphate phases may consume OH⁻ ions, further contributing to pH reduction. Importantly, the final pH remains within a physiologically acceptable range, supporting scaffold biocompatibility. The pH values of PBS controls without scaffolds remained stable throughout the incubation period. To support this observation, the pH variation of the PBS control over 28 days is presented as an inset in Fig 2b, demonstrating negligible fluctuations within the physiological range. Overall, the controlled mass loss and moderate pH shift demonstrate a favorable degradation profile that aligns with natural bone regeneration. However, in general, precise tuning of the PCL:BCP ratio is considered critical to balance mechanical integrity and degradation rate in polymer/ceramic composite scaffolds, given PCL’s inherently slow hydrolysis in PBS [3,35]. The observed pH decrease is consistent with prior reports. For example, Ananth et al. [36] reported that BCP scaffolds in Tris–HCl buffer exhibited a pH drop from 7.4 to 6.6 within 60 h due to Ca^2+^ and PO_4_^3−^ release. This confirms that BCP dissolution universally induces acidification, underscoring the need to optimize composition to prevent excessive pH shifts.

### 3.3. Bioactivity

**Fig 3:**
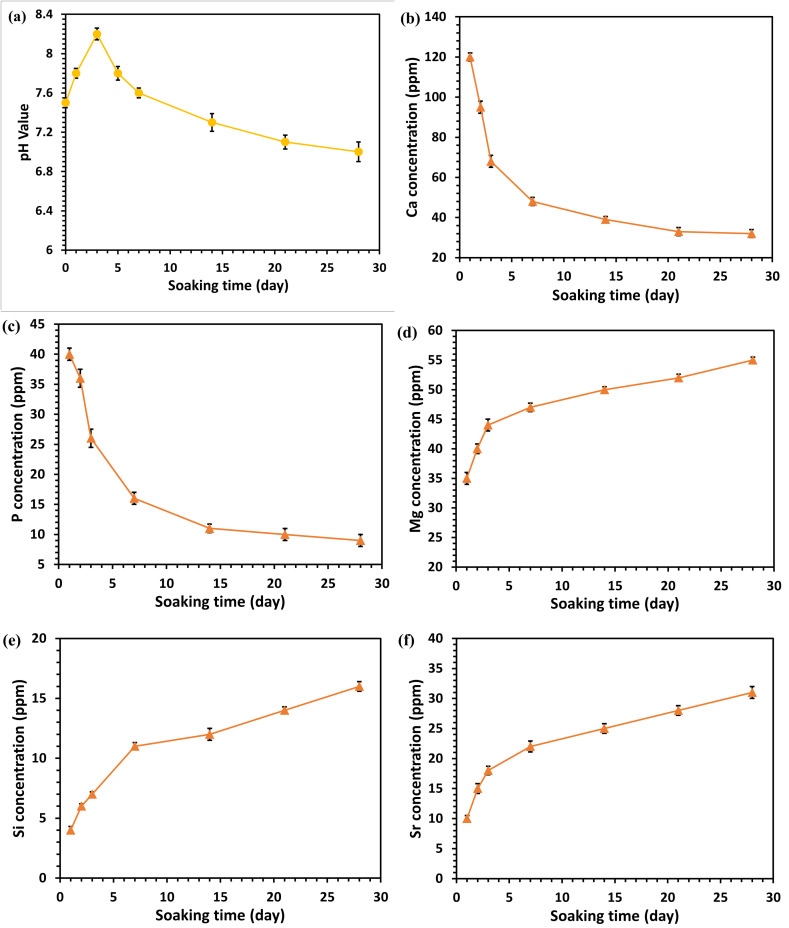
In vitro bioactivity and ion-release profile of PCL/40 wt.% ion-doped BCP scaffolds in SBF. (a) pH variation during a 28-day immersion of PCL/40 wt.% ion-doped BCP scaffolds in SBF; (b–f) concentrations of released ions, including Ca, P, Mg, Si, and Sr, from PCL/40 wt.% ion-doped BCP scaffolds over the same period.

The mechanism of apatite layer formation proceeds as follows: upon immersion of a porous scaffold in SBF, a rapid ion exchange occurs between the scaffold and the physiological medium. During this process, Ca^2+^ cations in the SBF are attracted to hydroxyl (OH⁻) and phosphate (PO₄³⁻) groups present on the scaffold surface. Consequently, the surface becomes positively charged, which in turn promotes the subsequent adsorption of negatively charged ions (OH⁻ and PO₄³⁻) from the solution, thereby initiating the formation of a dense apatite layer. The abundance of hydroxyl groups on the surface of inorganic calcium phosphate scaffolds further facilitates the attraction of cations from the medium. Through the repetition of these steps, a continuous hydroxyapatite layer is progressively established on the scaffold surface [37]. To evaluate the formation of an apatite layer on the scaffold surfaces *in vivo* as an indicator of the bioactivity of the fabricated scaffolds, they were immersed in SBF at ~ 37 °C [38]. Fig 3a illustrates the variation in pH of the SBF solution over immersion periods of 1, 3, 5, 7, 14, 21, and 28 days. In the initial stage (0–3 days), the pH increased from 7.4 to 8.2. However, after day 3, the pH gradually decreased, reaching approximately 7 at day 28. The pH values of SBF without scaffolds were stable throughout the experimental period. The partial dissolution of the surface layers on immersion in SBF and release of Si ions from the scaffold may lead to the initial increase in pH and the subsequent formation of apatite deposits, with the consumption of OH^-^ ion, leading to the decrease in pH [39]. The deposition of calcium and phosphate ions from the SBF onto the scaffold surface, leading to the nucleation and growth of an apatite layer, appears to mitigate the initial alkalinization of the medium. Overall, these pH fluctuations indicate the ability of the scaffold to interact with the biological environment and to facilitate apatite nucleation and growth. Earlier studies have shown that silicon-substituted hydroxyapatite exhibits an initial rise in SBF pH due to Ca^2+^ release and proton consumption during hydrated silica formation, followed by a decrease as calcium phosphate precipitates and matures into apatite. This behavior underscores the role of Si–OH groups in enhancing apatite nucleation and bioactivity [40].

In this study, the immersion assay was also performed to investigate the ion-release of PCL/40 wt.% ion-doped BCP scaffolds in the SBF solution. The concentrations of Ca, P, Mg, Si, and Sr ions were quantitatively assessed via ICP-OES, with the corresponding results depicted in Fig 3b to 3f. The concentration of Ca^2+^ exhibited a marked decrease from approximately 120 ppm at day 0 to nearly 30 ppm at day 28, accompanied by a concomitant decline in phosphorus levels from ~40 ppm to ~10 ppm (Fig 3b-c). The pronounced reduction, particularly within the first 7 days, can be ascribed to the rapid precipitation of calcium and phosphate ions, thereby facilitating the nucleation and subsequent formation of an apatite layer on the scaffold surface [41]. Beyond day 14, the rate of decrease slowed, approaching equilibrium, suggesting that apatite deposition had reached near completion. Fig 3d-f presents the ion release behavior of Mg, Si, and Sr, showing continuous increases throughout immersion. Mg concentration increased from ~34 ppm to ~53 ppm, Si from ~3 ppm to ~15 ppm, and Sr from ~9 to ~29 ppm. Si ion release and calcium phosphate deposition have been reported to increase under higher pH conditions [41]. The sustained release of these ions from the scaffold matrix indicates the progressive dissolution of the bioactive components embedded within the construct. Beyond enhancing bioactivity, these ions serve as potent nucleation promoters for apatite formation. Sr contributes to the stimulation of osteogenic processes and reinforces apatite deposition, while Mg plays a pivotal role in improving biocompatibility and regulating the morphology of apatite crystals. Extensive investigations have highlighted the role of Mg^2+^ ions in enhancing bioactivity, indicating that even trace levels can substantially enhance in vivo bone formation and promote osteoblast adhesion on glass surfaces. Specifically, incorporation of MgO up to approximately 17 wt% within the MgO–3CaO·P₂O₅–SiO₂ glass system has been shown to facilitate the formation of a calcium phosphate-enriched layer, thereby significantly accelerating surface mineralization upon immersion in SBF [41]. Si, as a bioactive element, accelerates apatite formation and contributes to the mechanical robustness of the deposited layer. The gradual increase in ion concentrations confirms that the scaffold exhibits not only pronounced bioactivity but also controlled solubility [42]. Previous studies have demonstrated that BCP scaffolds composed of 63.9% HA and 36.1% β-TCP, when coated with a Sr-, Mg-, and Zn-doped sol-gel-derived bioactive glass, exhibited enhanced bioactivity in SBF as well as improved compressive strength, thereby successfully achieving the intended functional outcomes [42]. The present results regarding the release of Ca, Sr, and P ions in SBF align well with the data previously reported by Merani et al. [43].

**Fig 4:**
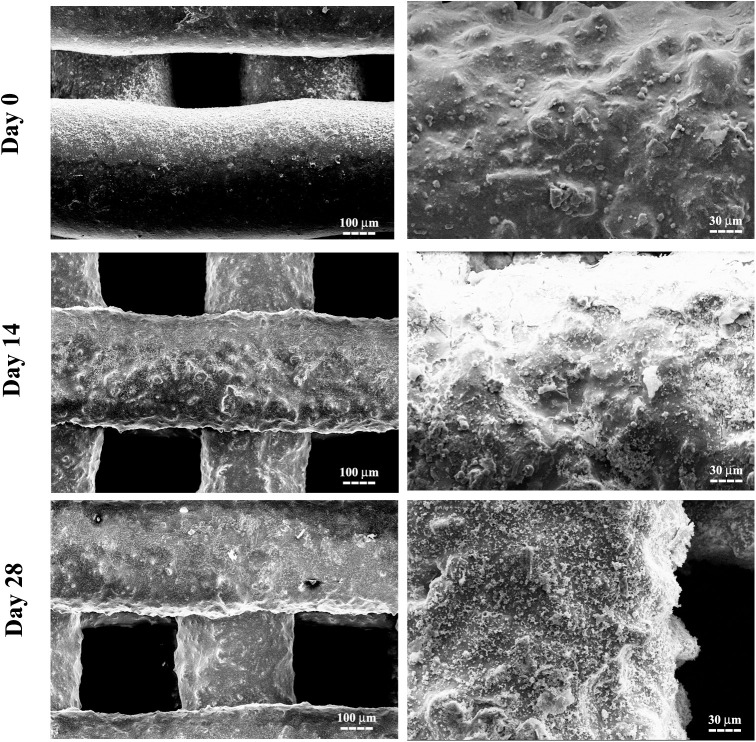
SEM images of the surface of the PCL/40 wt.% ion-doped BCP scaffold samples at two different magnifications after immersion in SBF for 0, 14, and 28 days.

The morphological analysis of the scaffold surfaces was performed using SEM to evaluate apatite mineralization. Fig 4 presents SEM images of the surface morphology of the PCL/40 wt.% ion-doped BCP scaffold samples before and after immersion in SBF at different time points. At day 0, the scaffold surface appeared relatively smooth. By day 14, the formation of initial apatite nuclei was evident, resulting in increased surface roughness and confirming ion absorption and the onset of mineralization. By day 28, a thicker and more homogeneous apatite layer had formed, indicating high bioactivity and successful apatite co-precipitation on the scaffold surface. Scaffold porosity enhances the surface area in contact with the solution, accelerating ion adsorption and promoting uniform apatite layer formation. Furthermore, the gradual biodegradation of PCL under physiological conditions provides space for new bone ingrowth, supporting tissue regeneration. The slow dissolution kinetics also prevent abrupt nanoparticle release, ensuring sustained BCP–SBF interactions and continuous apatite nucleation, thereby maintaining the scaffold’s bioactivity over time [44]. SEM images clearly show that the scaffold can form an apatite layer and interact with the biological environment.

### 3.4. SEM analysis of ZIF-8 coated scaffolds

**Fig 5:**
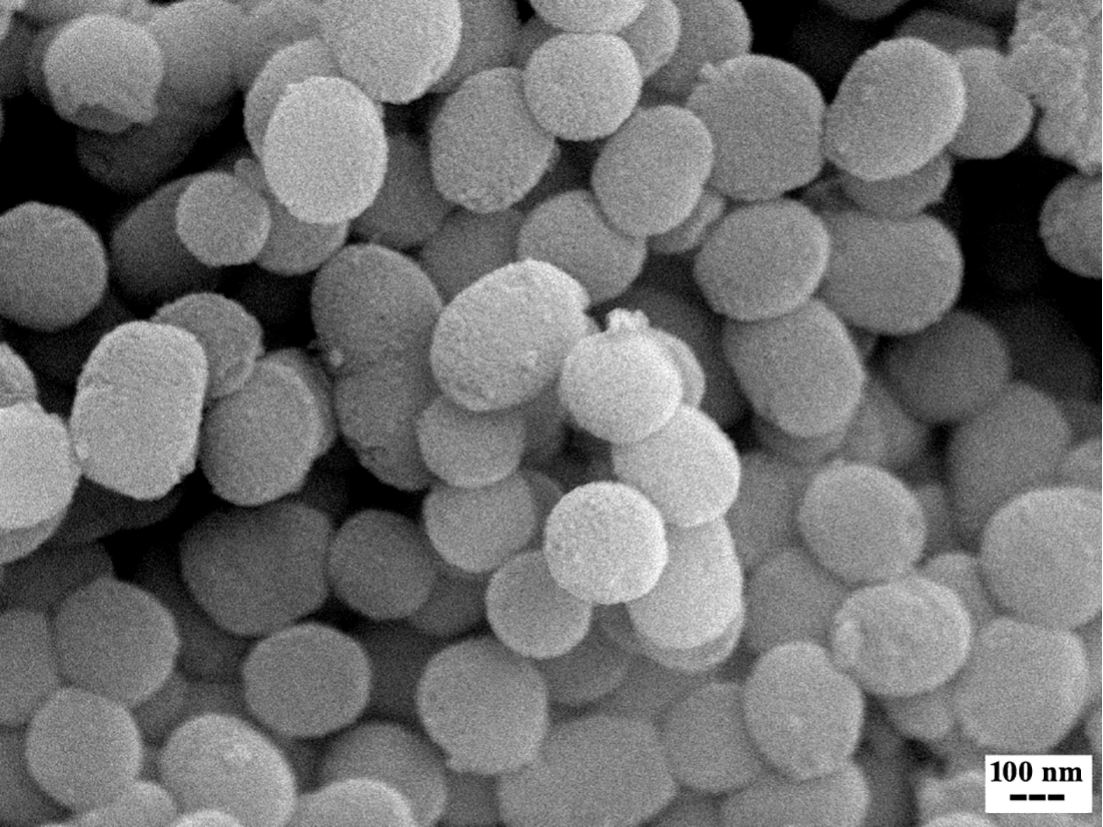
SEM image of a ZIF-8-coated PCL/40 wt.% ion-doped BCP scaffold.

The SEM images of the ZIF-8-coated PCL/40 wt.% ion-doped BCP scaffold (Fig 5) show a uniform coating of ZIF-8 nanoparticles on the scaffold surface. The nanoparticles are predominantly spherical, with average diameters below 200 nm, demonstrating the success of the in-situ crystallization process. Their narrow size distribution and morphological uniformity yielded a homogeneous coating across the scaffold surface, confirming effective reaction control. Such uniform ZIF-8 deposition not only enhances scaffold bioactivity and antibacterial capacity but also provides an interconnected porous architecture that facilitates nutrient transport and promotes favorable osteogenic interactions. Recent studies have demonstrated that in-situ growth of ZIF-8 yields homogeneous, densely packed nanoparticulate coatings on calcium-phosphate substrates, as evidenced by SEM/TEM imaging [18,45,46]. These reports corroborate our observations of uniform ZIF-8 distribution and provide methodological precedent for in-situ deposition on HA/BCP surfaces.

### 3.5. Cell studies

#### 3.5.1. Cell viability assessment scaffolds.

**Fig 6:**
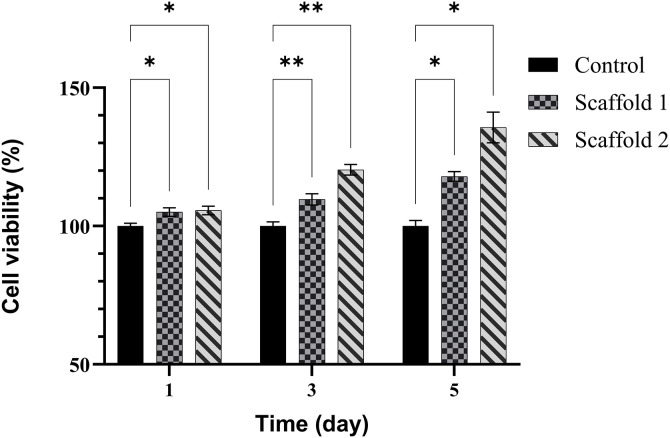
Viability of MG-63 cells cultured on the PCL/40 wt.% ion-doped BCP scaffold (Scaffold 1) and the ZIF-8–coated PCL/40 wt.% ion-doped BCP scaffold (Scaffold 2) after 1, 3, and 5 days. The control represents cells cultured in the absence of scaffolds. Data are presented as mean ± SD (n = 3), *p < 0.05, **p < 0.01.

**Fig 7:**
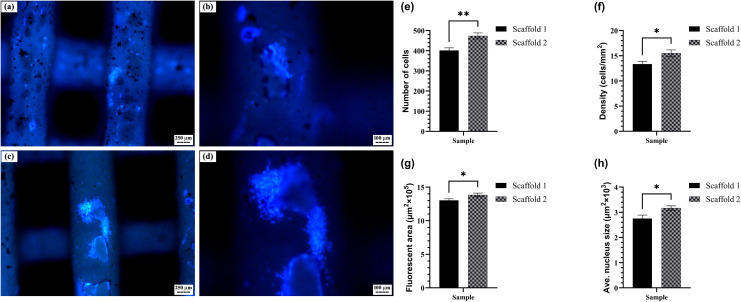
Fluorescence microscopy images of MG-63 cells cultured on(a, b) Scaffold 1 and (c, d) Scaffold 2 after 3 days of incubation, stained with DAPI to visualize cell nuclei at two different magnifications. **(e–h)** Quantitative image analysis of fluorescence images showing (e) number of cells, (f) cell density, (g) fluorescence area, and (h) average nuclear size. Data were obtained from randomly selected fields and analyzed using ImageJ software. Error bar represents SD (n = 3); *p < 0.05, **p < 0.01.

The viability and proliferation of MG-63 cells cultured on ZIF-8-coated PCL/40 wt.% ion-doped BCP scaffold (Scaffold 2) and uncoated PCL/40 wt.% ion-doped BCP scaffold (Scaffold 1) were evaluated using the MTT assay. Fig 6 illustrates the viability of MG-63 cells on Scaffolds 1 and 2 over 5 days. On day one, cell viability across all three groups, Scaffold 1, Scaffold 2, and the control, was nearly identical, indicating successful cell attachment to the scaffold surfaces and the absence of any initial cytotoxicity. As shown in Fig 6, cell viability on Scaffold 2 was significantly higher than that of both Scaffold 1 and the control (p < 0.05). This enhancement highlights the positive influence of ZIF-8 incorporation on cell adhesion and proliferation. By day five, Scaffold 2 continued to exhibit the highest cell viability, with statistically significant differences compared to both Scaffold 1 and the control. The progressive increase in cell viability demonstrates the scaffold’s ability to sustain proliferation and provide a favorable microenvironment for cellular growth. While Scaffold 1 also maintained acceptable cytocompatibility, its performance remained slightly superior to the control yet consistently lower than that of the ZIF-8–modified counterpart. The presence of ZIF-8 nanoparticles likely augmented scaffold bioactivity by promoting stronger cell–scaffold interactions. Consequently, Scaffold 2 not only supported initial adhesion but also effectively facilitated sustained cellular proliferation (Fig 7). Overall, both scaffolds displayed good biocompatibility, with no evidence of cytotoxic effects throughout the experimental period. Nevertheless, incorporation of ZIF-8 markedly enhanced adhesion, proliferation, and viability, positioning the PCL/BCP/ZIF-8 scaffold (Scaffold 2) as a good candidate for BTE applications due to its improved bioactivity and cell-supportive capacity [22]. These findings are in line with comprehensive reviews indicating that ZIF-8, along with other MOF-based biomaterials, can markedly enhance cellular adhesion, proliferation, osteogenic differentiation, and apatite mineralization, while also highlighting the potential cytotoxic effects of excessive Zn^2+^ release [47]. Comparable results have been reported by Shuai et al. [48], where ZIF-8 coatings on HA and polymer-based scaffolds enabled controlled Zn^2+^ release, promoted apatite mineralization, and significantly improved biocompatibility, thereby corroborating the dual role of ZIF-8 as both a bioactive ion reservoir and a surface-enhancing agent. It should be noted that viability values above 100% reflect increased metabolic activity relative to the control rather than absolute cell numbers. The culture medium was refreshed regularly to maintain physiological pH, and scaffold-only blanks were used to eliminate potential interference with the MTT assay.

#### 3.5.2. Analysis of phalloidin and DAPI test.

**Fig 8:**
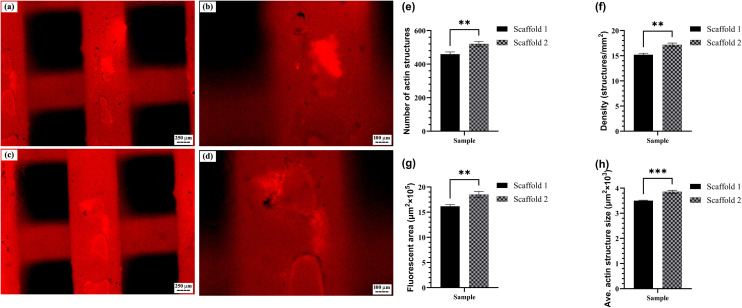
Fluorescence microscopy images of MG-63 cells cultured on(a, b) Scaffold 1 and (c, d) Scaffold 2 after 3 days of incubation, stained with FITC-phalloidin to visualize the actin cytoskeleton at two different magnifications. **(e–h)** Quantitative image analysis of fluorescence images showing (e) number of actin structures, (f) actin structure density, (g) fluorescence area, and (h) average actin structure size. Data were obtained from randomly selected fields and analyzed using ImageJ software. Error bar represents SD (n = 3); **p < 0.01, ***p < 0.001.

MG-63 cells were seeded on Scaffold 1 (without ZIF-8) and Scaffold 2 (with ZIF-8) for 3 days and stained with DAPI (nuclei) and FITC-phalloidin (actin cytoskeleton). Fluorescence images at two different magnifications, as well as the corresponding quantitative analysis results, are presented in Figs 7 and 8. DAPI staining revealed moderate cell attachment on Scaffold 1 with somewhat clustered nuclei (Fig 7a and 7b), whereas Scaffold 2 showed significantly higher and more uniformly distributed nuclei (Fig 7c and 7d). Quantitative analysis indicated greater cell number (~470 vs. ~ 400; p < 0.01), higher density (~15 vs. ~ 13 cells/mm^2^; p < 0.05), increased total fluorescent nuclear area (p < 0.01), and larger average nuclear size (p < 0.05) on Scaffold 2 (Fig 7e to 7h). Higher magnification confirmed more elongated, healthy nuclear morphology on Scaffold 2 compared to Scaffold 1.

FITC-phalloidin staining displayed sparse, disorganized actin on Scaffold 1 (Fig 8a and 8b) versus extensive, dense, and aligned actin structures on Scaffold 2 (Fig 8c and 8d), indicative of superior spreading and cytoskeletal organization. Quantification showed higher actin structure count (~520 vs. ~ 460; p < 0.01), density (~17 vs. ~ 15 structures/µm^2^; p < 0.01), total fluorescent area (p < 0.01), and average structure size (p < 0.001) on Scaffold 2 (Fig 8e to 8h). This improvement is likely attributable to the presence of ZIF-8 nanoparticles, which provided a more favorable microenvironment for cell adhesion and proliferation. The incorporation of ZIF-8 enhanced scaffold–cell interactions, supporting not only cell adhesion but also cytoskeletal development, likely due to its bioactive and antibacterial properties. Overall, while both scaffolds supported cell adhesion and growth, the presence of ZIF-8 amplified these responses. The ZIF-8–coated scaffold exhibited superior biocompatibility and appears particularly promising for bone tissue engineering applications where enhanced cellular interaction is critical [22]. Our observations regarding improved cellular adhesion, spreading, and proliferation on ZIF-8-modified scaffolds are consistent with findings by Wang et al. [18] and Zou et al. [49], who demonstrated enhanced cell attachment and cytoskeletal organization via DAPI and phalloidin staining upon ZIF-8 functionalization.

#### 3.5.3. Analysis of Alizarin red staining.

**Fig 9:**
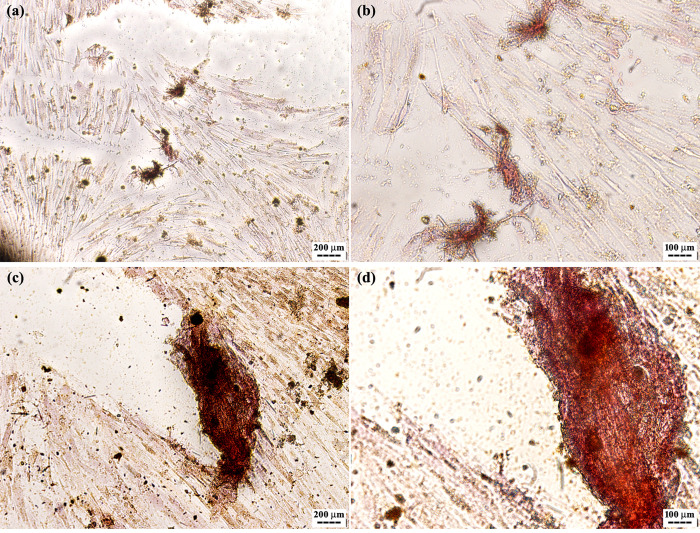
Optical images of Alizarin Red staining of hBMSCs cultured on: **(a, b)** Scaffold 1 and **(c, d)** Scaffold 2 after 14 days, shown at two different magnifications.

Osteoblastic differentiation is commonly assessed by calcium deposition, the main mineral component of the bone matrix, typically evaluated using Alizarin Red staining [50]. In Scaffold 1, Fig 9a and 9b show that mineral deposits appear as small, scattered clusters on the scaffold surface. The relatively low red intensity indicates limited mineralization, primarily localized to specific sites, suggesting that the uncoated scaffold has a restricted capacity to induce extracellular matrix mineralization, likely due to insufficient release of bioactive ions. In contrast, the ZIF-8–coated scaffold (Fig 9c and 9d) exhibits larger and denser mineral clusters. The markedly higher red intensity indicates enhanced mineralization compared to the uncoated scaffold, likely resulting from the controlled and synergistic release of bioactive ions—Ca^2+^, P^5+^, Mg^2+^, and Si^4+^ from the BCP phase, and Zn^2+^ from the ZIF-8 coating—which collectively stimulate osteogenic differentiation and improve the scaffold’s overall bioactivity.

It should be noted that matrix mineralization was quantified using Alizarin Red S staining, which is the standard method for assessing calcium deposition in osteogenic differentiation. However, given the presence of Mg^2+^ and Sr^2+^ in the ion-doped BCP, potential minor binding to these divalent cations cannot be entirely ruled out. Future studies could benefit from complementary von Kossa staining (specific for phosphates) to confirm the nature of the mineral deposits.

**Fig 10:**
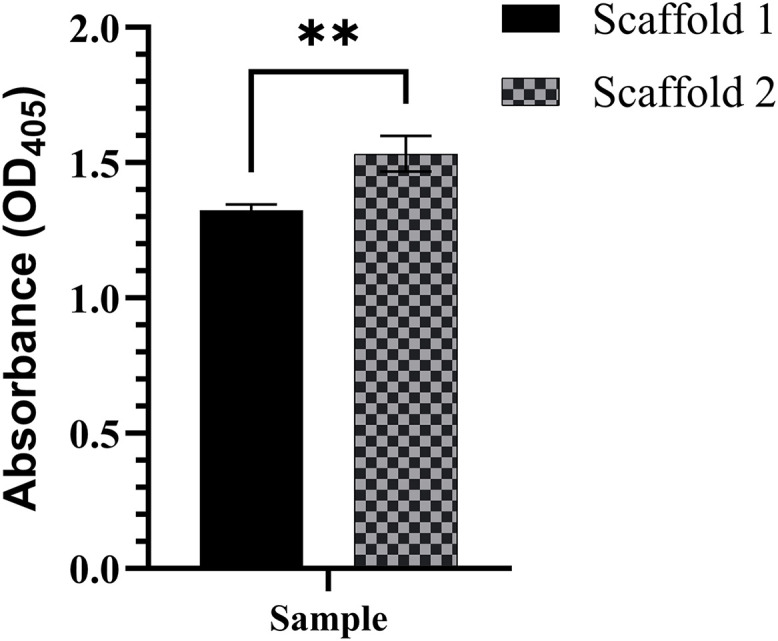
Quantitative Alizarin Red staining of hBMSCs cultured on Scaffold 1 and Scaffold 2 after 14 days. Data are mean ± SD (n = 3). **p < 0.01 (unpaired Student’s t-test).

For quantitative assessment, the absorbance of the extracted Alizarin Red S solution was measured at 405 nm (Fig 10). Scaffold 1 exhibited lower absorbance (1.32 ± 0.05), reflecting limited mineralization, whereas Scaffold 2 showed significantly higher absorbance (1.53 ± 0.08; p < 0.01), indicating enhanced extracellular matrix mineralization. This enhancement is likely attributable to the positive effect of the ZIF-8 coating on bioactive ion release and overall scaffold bioactivity. Both qualitative and quantitative analyses clearly demonstrated that the ZIF-8 coating markedly promotes extracellular matrix mineralization. This effect is primarily due to the controlled release of bioactive ions: Sr^2+^, which stimulates osteoinduction and osteoblastic differentiation; Mg^2+^, which modulates the organization of the mineralized matrix; and Si^4+^, which accelerates mineral deposition. Collectively, ZIF-8–mediated enhancement of surface bioactivity creates an optimal microenvironment for robust cell adhesion and dynamic interactions with the extracellular matrix [3]. These results align with previous reports showing that mineralized ZIF-8 can induce osteogenic differentiation even in the absence of osteogenic supplements. Specifically, Alizarin Red–positive nodule formation and upregulation of osteogenic markers (ALP, Runx2, OCN, and OPN) at both gene and protein levels confirm the potent stimulatory effect of ZIF-8 on mineralization and osteoblast differentiation [18].

## 4. Conclusion

This study successfully developed 3D-printed PCL/BCP composite scaffolds (40 wt.% ion-doped BCP with Sr^2+^, Mg^2+^, Si^4+^) via the FDM method, further functionalized with in situ ZIF-8 coating. The optimized formulation achieved excellent mechanical performance (compressive strength: 29.7 MPa; modulus: 0.41 GPa), surpassing pure PCL and other BCP ratios, making it suitable for load-bearing bone defects. Controlled degradation (~8% mass loss over 28 days) and stable pH in PBS ensured biocompatibility, while immersion in SBF demonstrated high bioactivity through rapid apatite formation and sustained release of Ca^2+^, P, Mg^2+^, Si^4+^, and Sr^2+^ ions. SEM confirmed uniform ZIF-8 nanoparticle deposition (<200 nm), enhancing surface bioactivity. In vitro evaluations using MG-63 cells and hBMSCs revealed that ZIF-8-coated scaffolds significantly improved cell adhesion, proliferation (MTT), cytoskeletal spreading (DAPI/phalloidin), and extracellular matrix mineralization (Alizarin Red S) compared to uncoated controls (p < 0.05), confirming superior osteogenic induction. The synergistic combination of ion-doped BCP, 3D printing precision, and ZIF-8 biofunctionalization created a mechanically robust, biodegradable, and highly osteoinductive platform, addressing key limitations of traditional PCL and ceramic scaffolds. This system supports vascularization, nutrient diffusion, and gradual tissue replacement, positioning it as a promising candidate for advanced bone regeneration.

Limitations of this study include the absence of in vivo validation, which is critical to assess long-term biocompatibility, inflammatory response, and osseointegration under physiological loading. Mechanical testing was conducted under static conditions; dynamic fatigue and creep behavior in body fluid remain unexplored. Additionally, while Zn^2+^ release was controlled, dose-dependent cytotoxicity at higher concentrations was not fully investigated. So, Future work should include animal models (e.g., rat femoral or calvarial defects) to evaluate bone formation, angiogenesis, and scaffold resorption in vivo. Long-term mechanical stability under cyclic loading, combined with histological and micro-CT analysis, is essential. Incorporating growth factors (e.g., BMP-2) within ZIF-8 pores or vascular endothelial cells could further enhance osteogenesis and vascularization. Clinical translation will require scalability, sterilization compatibility, and regulatory compliance studies.
