# Supplementary material for: ZIF-8-coated 3D-printed PCL/ion-doped BCP scaffolds for enhanced bone regeneration
Source: PLoS One. 2026 Jun 11;21(6):e0347048. doi: 10.1371/journal.pone.0347048 (PMC13257968; doi:10.1371/journal.pone.0347048)
Supplement: Attachment — Submitted filename: Response_R1.docx [file pone.0347048.s002.docx]

Manuscript Number: **PONE-D-25-64571**

**Dear Editor**

We would like to express our sincere appreciation for your valuable comments and those of the reviewers regarding our manuscript. The insights provided were exceptionally constructive and have significantly contributed to the improvement of the quality and clarity of our work. In the revised version, we have carefully addressed all points raised in the reviewers' comments. Each modification has been thoroughly explained in our point-by-point response and highlighted within the revised manuscript. To facilitate the review process, we have also included a tracked version of the manuscript at the end of this response letter, clearly showing all the revisions made. We hope that these modifications meet the expectations of the reviewers and the editorial board, and that the manuscript is now suitable for publication in *PLOS ONE*.

Sincerely,

**Ghasem Dini (Ph.D.)**

Department of Nanotechnology

Faculty of Chemistry

University of Isfahan

Isfahan 81746-73441, Iran

g.dini@sci.ui.ac.ir

Tel.: +983137934914

Fax: +983137932700

**Reviewer #1:**

The authors describe the fabrication of PCL/BCP scaffolds for bone regeneration applications. They investigated the effect of varied Sr, Mg, Si ion doping concentrations and presence of functional ZIF-8 coatings on material mechanical properties and bioactivity. The manuscript is clearly written, the methodology is well described, and the conclusions appear supported by the results.

1. For mechanical testing, what was the orientation of 3D printed sample with respect to the compression testing?
We appreciate the reviewer’s insightful comment. The loading orientation during compression testing has been explicitly specified in the revised manuscript. The scaffolds were compressed perpendicular to the printing layers to evaluate through-thickness mechanical properties. The corresponding clarification has been incorporated into the *Evaluation of Mechanical Properties* section.

2. For cell viability tests (Section 3.4.1, Figure 6)

a. What was the control sample? The manuscript simply states a non-scaffold sample. Tissue culture plastic? If so, how does the porosity (available surface area for cells to attach) of the control compare to scaffold samples and does this consideration impact your analysis?

Thank you for raising this important point. In our study, the control group consisted of MG-63 cells cultured under identical conditions in tissue culture wells without scaffolds (cells cultured without scaffolds), which corresponds to standard tissue culture plastic (TCP). This control was used as a baseline reference to confirm normal cell metabolic activity and to normalize the MTT data, following common practice in scaffold cytocompatibility studies.

We acknowledge that the available surface area and porosity may differ between the control and the scaffold samples. However, the primary objective of this study was not to perform a direct quantitative comparison between the control group and the two scaffold groups (ZIF-8–coated and uncoated), but rather to conduct a comparative evaluation between the PCL/BCP scaffolds with and without ZIF-8 coating. Since both scaffold groups were tested under identical experimental conditions and referenced to the same control, the influence of the control remains constant across the groups and therefore does not affect the relative comparison between them. Consequently, any differences observed in cell viability can be attributed to the presence or absence of the ZIF-8 coating rather than to the control conditions.

b. For Fig. 6, the convention is generally to draw bars between pairs to denote statistical significance in pair-wise tests, and the lack of bars indicates no statistically significant difference. In the text, authors make statements about statistical significance between Scaffolds 1 and 2. Please confirm the accuracy of the analysis in the manuscript or add bars to Fig 6 if appropriate.

Thank you for your careful observation. Statistical significance in Figure 6 has been indicated using bars/brackets between the compared groups according to standard convention. It is possible that these markings were not clearly visible in the submitted version due to formatting or resolution issues. We will recheck the figure and provide a revised high-quality version to ensure the statistical annotations are clearly presented.

3. Section 3.4.2 and Figure 7 present fluorescence imaging of cells on scaffolds with and without ZIF-8. The authors claim “A higher density of nuclei was observed on scaffold 2 (Fig. 7c) compared to scaffold 1 (Fig. 7a), indicating enhanced cytocompatibility” and “The scaffold 2 (Fig. 7d) exhibited more extensive and denser action filament distribution compared to the scaffold 1…” It is unclear how these findings were reached based on the data presented in Fig 7. Are these findings based on quantitative image analysis (cell counts? fluorescence intensity)?

We thank the reviewer for this valuable point and agree that the basis for our statements on nuclear density and actin filament distribution should be more explicitly linked to quantitative data. The claims of higher nuclear density and more extensive actin organization on Scaffold 2 are based on quantitative image analysis of the fluorescence micrographs, as shown in the bar graphs of Figures 7 and 8 (previously Figure 7). For DAPI (nuclear staining): Number of nuclei, cell density (nuclei/mm²), total fluorescent nuclear area, and average nuclear size were measured (Figure 7e–h). For FITC-phalloidin (actin cytoskeleton): Number of actin structures, density (structures/mm²), total fluorescent actin area, and average actin structure size were quantified (Figure 8e–h).

All parameters showed statistically significant improvements on Scaffold 2 (**p < 0.01 or ***p < 0.001 in most cases; unpaired Student's t-test), with data presented as mean ± SD from at least 5–10 fields of view per scaffold (n=3 independent replicates). To improve clarity, we have revised the text in Section 3.4.2. Additionally, we have expanded the Materials and Methods (Fluorescence Staining subsection) with a description of the quantification method.

4. Was statistical analysis performed in Fig 9? If so, please include the results of that analysis in the Figure.

We thank the reviewer for pointing this out. Yes, statistical analysis was performed on the data in Figure 10 (previously Figure 9). The comparison between Scaffold 1 and Scaffold 2 was evaluated using an unpaired Student's t-test, yielding p < 0.01 (indicated by ** in the figure). The statistical method is also explicitly mentioned in the Materials and Methods section.

**Reviewer #2:**

Khademi et al present a bone regeneration scaffold that has been 3D printed from polycaprolactone (PCL) and biphasic calcium phosphate (BCP) doped with Sr2+, Mg2+ and Si4+ cations. The scaffold was subsequently coated with a zeolitic imidazolate framework (ZIF-8) consisting if zinc ion and 2-methylimidazole. The mechanical properties of the scaffolds were investigated and cell culture studies were completed.

Overall, I find the work is worth publication. However, I believe it requires major revision prior to publication as an adequate discussion and literature context of the results obtained in this study are lacking. I recommend the authors address the following comments:

(1) On page 4 in Section 2.2.2, how long was the BCP-PCL mixture typically mixed until the solvent was fully evaporated? At what temperature? With magnetic stirring or shaking? Also how were the authors able to confirm that a "uniform solid composite" was obtained?

We sincerely thank the reviewer for their careful attention and insightful comments. In response to your query regarding the preparation of the BCP/PCL composite: BCP is mixed with the PCL solution and stirred until the solvent is completely evaporated. The exact evaporation time is not specified because, for example, fabricating a 35 wt% BCP scaffold requires a smaller volume of chloroform, whereas producing a 45 wt% BCP scaffold requires a larger volume. This is due to the need for more extensive sonication to achieve a uniform composite when a higher amount of powder is used. Consequently, the powder is dispersed in more chloroform to ensure homogeneity. Naturally, a larger volume of chloroform requires a longer evaporation time. The recommended procedure is to stir the BCP/PCL mixture at room temperature on a magnetic stirrer until the chloroform is fully evaporated, and the stirrer should not be turned off until complete evaporation is achieved. These clarifications have been incorporated into the revised manuscript.

To demonstrate the uniformity of the resulting composite, it can be stated that any minor heterogeneity in the composite would cause nozzle clogging during the printing process. We tested multiple fabrication methods, many of which resulted in nozzle blockage or ink flow issues, indicating non-uniformity of the composite. Although using TEM could be a suitable approach to verify uniformity, our practical experience demonstrates that the described fabrication process reliably produces a homogeneous composite. Some modifications were made to Section 2.2.2

(2) On page 5 in Section 2.2.4, for the degradation evaluation, what was the control scaffold? Just PCL? And why was degradation only studied in PBS? Wouldn't at least cell culture medium be more representative or the SBF? I would recommend repeating the assay in one or both.

We thank the reviewer for this insightful comment. The degradation assay was designed to evaluate the intrinsic hydrolytic biodegradability of the PCL/BCP scaffolds using a gravimetric approach. For each time point, measurements were performed in triplicate (n = 3) to ensure reproducibility. Although gravimetric degradation studies do not strictly require a separate control, pure PCL scaffolds were included as a reference to contextualize the degradation behavior of the composite scaffolds.

Regarding the choice of immersion medium, phosphate-buffered saline (PBS) was selected because it is widely used as a standard medium for in vitro degradation studies of polymer-based and polymer/ceramic composite scaffolds. PBS provides a controlled environment with physiological pH and ionic strength, allowing isolation of hydrolytic degradation mechanisms without interference from additional variables [e.g., Woodruff & Hutmacher, Prog. Polym. Sci., 2010; Kharaziha et al., Acta Biomater., 2014].

While simulated body fluid (SBF) or cell culture media may better mimic physiological conditions, their use in gravimetric degradation assays can complicate data interpretation. In particular, immersion in SBF is known to promote surface mineralization and apatite layer formation on bioactive scaffolds, which can result in an apparent increase in scaffold weight rather than true mass loss. Similarly, cell culture media introduce additional factors such as protein adsorption, ion exchange, and enzymatic activity. These effects may mask the intrinsic degradation behavior of the polymeric matrix and confound quantitative weight-loss measurements. Therefore, PBS was chosen as an appropriate first-line medium to assess the intrinsic degradation kinetics of the scaffolds.

(3) On page 6 in Section 2.4.1, how was blanking done for the cell viability assay?

We appreciate the reviewer’s insightful comment. Cells cultured without scaffolds served as the control.

(4) On page 7 in Section 3.1, I do not understand the criteria for choosing the 40wt% BCP version. What mechanical properties/profile would be optimal for bone grafting and osteocyte differentiation? Such cell types are sensitive to substrate mechanical properties. What does the literature say, how do these results compare to other scaffolds previously presented or used clinically?

Thank you for your insightful question regarding the choice of the PCL/BCP composition with 40 wt% BCP. As a criterion for selection, we considered the mechanical properties in relation to native bone tissue. The values of 0.41 GPa for modulus, 8.9 MPa for yield strength, and 29.7 MPa for compressive strength are very close to the range reported for human trabecular/spongy bone. In other words, these properties overlap with the bone found in joints, the ends of long bones (such as the epiphyses of the femur or tibia), and the inner parts of the bone structure (trabecular/cancellous bone). The mechanical profile of the 40 wt% BCP scaffold — compressive modulus (~0.41 GPa), yield strength (~8.9 MPa), and ultimate compressive strength (~29.7 MPa) — falls within the range of values reported for human trabecular (cancellous) bone. Literature indicates that cancellous bone exhibits elastic moduli typically on the order of 0.1–2 GPa and compressive yield strengths in the range of ~1–10 MPa, with ultimate strengths varying depending on anatomical site and density. Given this correspondence, the 40 wt% BCP composition was selected because it provides a mechanical profile closer to that of trabecular/spongy bone, offering a more representative substrate mechanical environment for potential load-bearing and bone regeneration applications, compared to other compositions tested (e.g., 35 wt% or 45 wt% BCP). This alignment with native cancellous bone mechanics supports the scaffold’s suitability in bone tissue engineering, where matching the local trabecular mechanical environment can influence cell behavior and osteogenic responses. The correction was made to the introduction section on page 3, lines 24 to 29.

(5) On page 8 in Section 3.2, the authors mention that a "mildly acidic microenvironment" should "enhance osteoblast activity and bone remodeling." What pH range would acceptable for this? Are there references to support this?

Thank you for the valuable comment. The sentence suggesting a potential stimulatory effect of mildly acidic pH on osteoblast activation and osteogenic potential has been removed. Although slight pH variations may influence cellular behavior, the current literature does not provide sufficiently consistent evidence to directly support enhanced osteogenesis under acidic conditions. Therefore, to avoid overinterpretation and ensure scientific accuracy, this statement was omitted.

(6) On page 8 in Section 3.2, the authors indicate that the "PBS controls without scaffolds showed stable pH throughout the study." I would highly recommend adding this data to supplementary information.

We thank the reviewer for this helpful suggestion. The pH variation of the PBS control over the 28-day incubation period is presented as an inset in Figure 2b.

(7) On page 8 in Section 3.2 lines 15-16, the authors mention that the PCL:BCP ratio is critical to degradation rate. Were other BCP levels investigated other than 40% BCP for degradation?

We thank the reviewer for this important question. In the present study, degradation behavior was systematically evaluated only for the nanocomposite scaffold containing 40 wt% BCP. Although scaffolds with 35 and 45 wt% BCP were fabricated and mechanically characterized, they were not further investigated for degradation. As clarified in the revised Introduction, the primary objective of this work was to achieve an optimal balance of mechanical performance suitable for the intended application. Once the target mechanical integrity was achieved at 40 wt% BCP, the other compositions no longer satisfied the required mechanical criteria and were therefore excluded from further degradation analysis.

We would like to emphasize that the statement regarding the critical role of precise tuning of the PCL:BCP ratio in balancing mechanical integrity and degradation rate is intended as a general design consideration, supported by established literature on polymer/ceramic composite scaffolds, rather than a direct conclusion drawn from comparative degradation data of multiple BCP contents within this study. The manuscript has been revised to clarify this point and avoid potential misinterpretation.

In addition, were the mechanical properties of the various BCP level scaffolds evaluated during degradation? This could be included in SI.

We thank the reviewer for this valuable suggestion. We agree that evaluating the evolution of mechanical properties during degradation and immersion in PBS would provide additional insight into the long-term performance of the scaffolds. However, such time-dependent mechanical characterization was not conducted in the present study. As clarified in the manuscript, the mechanical properties were evaluated in the as-fabricated state to identify the composition that meets the required mechanical criteria for the intended application. Once the optimal composition was identified, the degradation behavior was investigated to assess its intrinsic hydrolytic stability. The evolution of mechanical properties during degradation was therefore beyond the scope of this study and has been acknowledged as a limitation in the discussion of mechanical properties.

(8) On page 9 in Section 3.3, for the immersion assay in SBF, were controls without Mg, Si and Sr cations completed?

We thank the reviewer for raising this important point regarding controls in the SBF immersion assay. The decision was based on our previous work [33], which already demonstrated the beneficial osteogenic effects of these doped ions (Sr²⁺ 5.0 mol%, Mg²⁺ 0.6 mol%, Si⁴⁺ 0.2 mol%) in promoting apatite formation, cell proliferation, and differentiation compared to undoped systems. The present study builds on that foundation by evaluating how ZIF-8 further enhances the bioactivity of the already optimized ion-doped formulation.

And in Figure 3 (f) on page 11 the description mentions PCL + 40% BCP "including Ca, P, Mg, Si and Sr". Do the authors mean ZIF-8 - PCL+ 40% BCP?

No. We only used ZIF-8 for the bioassays on the nanocomposite scaffolds. The text has been revised for clarity. In addition, the caption of Fig. 3 has been modified for clarity.

(9) On page 11 in Section 3.3, lines 12 - 17 would be good information for the introduction.

Thank you for the suggestion. The information has been moved to the Introduction as recommended.

(10) On page 12 in Section 3.5, lines 7-8 discuss the shape/morphology and sizes of the ZIF-8 nanoparticles, giving sizes below 300 nm for the nanoparticles. And the authors present Figure 5 with a SEM image. I do not find this image sufficient to quote a size below 300 nm for the nanoparticles. The authors should include images showing more detail of the nanoparticles at a higher magnification to clearly demonstrate the morphology and sizes of the particles.

We thank the reviewer for the comment. A new SEM image at higher magnification has been included, and the average particle size is now reported as below 200 nm.

(11) On page 13 in Section 3.4.1, in lines 11 -12 the authors mention that scaffold 2 has a higher cell viability than scaffold 1. There is no direct statistical comparison from the data in Figure 6 for scaffold 1 and 2. Additionally, the cell viabilities indicated for scaffolds 1 and 2 are significantly above 100%, starting at day 3. At day 3 in the degradation and bioactivity studies, there was a pH spike to higher pH and by day 5, the pH is lower than at day 0/1. Viability assay signals can be enhanced by various factors. pH changes can have an impact on cell viability assays. In addition, reagents such as MTT can also impact the results. I would recommend controls as similar pH as the samples with scaffold to rule out pH effect on cell viability.

We thank the reviewer for this valuable and insightful comment. First, regarding the statistical comparison between scaffold 1 and scaffold 2, the original figure presents a direct statistical analysis of these two groups. Data were analyzed using one-way ANOVA followed by Tukey’s post hoc test, confirming that scaffold 2 exhibits significantly higher cell viability than scaffold 1 on days 3 and 5 (p < 0.05).

Second, concerning viability values exceeding 100%, the MTT results were normalized to the control group at the corresponding day, defined as 100%. Values above 100% indicate enhanced metabolic activity and cell proliferation rather than absolute viability, consistent with previous scaffold-based cytocompatibility studies.

We acknowledge that environmental factors such as pH fluctuations may influence colorimetric viability assays. Although a temporary pH increase was observed during degradation studies, the cell culture medium in the experiments was regularly refreshed and buffered (DMEM with bicarbonate/CO₂ system), maintaining physiological pH (7.2–7.4). Therefore, significant pH-related interference with the MTT assay is unlikely.

Furthermore, blank controls containing scaffolds without cells were included to exclude possible interference from scaffold components or reagents with the MTT signal. This clarification has been added to the Methods section.

Following the reviewer’s suggestion, we have included an explanatory sentence in the revised manuscript discussing the potential influence of pH and assay conditions on viability measurements and the controls used. We thank the reviewer for this constructive suggestion, which improved the clarity and reliability of our results.

(12) On page 15 Figure 7, the digital version of the images seem to indicate that the substrate is also absorbing the fluorescent stain. The background fluorescence on both DAPI and Phalloidin appear similar to the cells in the images. In addition, I would strongly recommend adding higher magnification images of the cells to show the cells better and allow the reader to investigate the cell morphology easier. It also might be beneficial to overlap the images with a live/dead imaging assay to confirm which cells are live and staining.

We thank the reviewer for highlighting potential background fluorescence in Figure 7 (now Figures 7 and 8 in the revised manuscript), the need for higher magnification, and the suggestion for live/dead assay. Although slight background fluorescence from the scaffold may be observed, quantitative analysis was performed exclusively based on nuclear staining, allowing a clear distinction between cells and the substrate. Importantly, identical staining and imaging conditions were applied to both scaffolds; therefore, any background signal was consistent and did not affect the comparative analysis. We applied uniform background subtraction (ImageJ rolling-ball algorithm) during analysis.

We agree that higher magnification is valuable for morphology assessment. New images have been added to the revised figures. These clearly show better spreading, elongated nuclei, and denser actin fibers on Scaffold 2. The figure legends have been updated to note the magnifications. Regarding the suggestion of live/dead staining, we agree that such assays provide complementary information; however, evaluation of cytocompatibility in the present study was sufficiently addressed through nuclear staining and quantitative image analysis within the scope of this work.

(13) On page 16 Figure 8, it is mentioned that the images are at 2 different magnifications. What were they? How many samples were observe for the presented images? Are they all from one sample per scaffold or multiple? And finally, Alzirin Red S binds to other divalent ions, such as Mg2+, Sr2+, etc. It might be a good idea to cross-check the results with von Kossa's protocol.

We thank the reviewer for these detailed questions regarding Figure 8 (FITC-phalloidin staining of actin cytoskeleton, now Figure 9) on page 16. The images are presented at two different magnifications. Panels (a) and (c) were acquired at low magnification to provide an overview of cell distribution and overall actin organization across larger scaffold areas. Panels (b) and (d) were acquired at higher magnification to show detailed cytoskeletal structures, stress fibers, and cell spreading/morphology. Panel (b) is the higher-magnification view corresponding to the region in panel (a) (Scaffold 1), and panel (d) corresponds to panel (c) (Scaffold 2).

The representative images shown are selected from multiple fields of view. For each scaffold type (Scaffold 1 and Scaffold 2), images were acquired from at least 5–10 randomly selected fields per sample. The presented images are typical and representative of the consistent observations across these replicates and fields.

Regarding Alizarin Red S specificity and suggestion for von Kossa staining: We appreciate the reviewer’s caution that Alizarin Red S (ARS) primarily chelates Ca²⁺ but can also bind other divalent cations such as Mg²⁺ and Sr²⁺ (which are present in our ion-doped BCP formulation). While ARS is widely accepted as the gold standard for quantifying matrix mineralization in osteogenic cultures (due to its strong affinity for calcium phosphates), potential cross-reactivity with other ions could theoretically influence staining intensity in ion-doped systems.

Unfortunately, we do not currently have access to perform von Kossa staining (which targets phosphate groups more specifically and is less prone to interference from non-calcium divalent ions) in our laboratory facilities. However, we fully agree that combining or cross-validating ARS with von Kossa would strengthen the mineralization data and better distinguish true calcium phosphate deposition.

To address this valid concern, we have added the following clarifying and cautionary statement to the revised manuscripts (Mineralization assessment):

"Matrix mineralization was quantified using Alizarin Red S staining, which is the standard method for assessing calcium deposition in osteogenic differentiation. However, given the presence of Mg²⁺ and Sr²⁺ in the ion-doped BCP, potential minor binding to these divalent cations cannot be entirely ruled out. Future studies could benefit from complementary von Kossa staining (specific for phosphates) to confirm the nature of the mineral deposits."

(14) On page 17 in Figure 9 was there a statistical significance found between Scaffold 1 and 2?

We thank the reviewer for pointing this out. Yes, statistical analysis was performed on the data in Figure 10 (previously Figure 9). The comparison between Scaffold 1 and Scaffold 2 was evaluated using an unpaired Student's t-test, yielding p < 0.01 (indicated by ** in the figure). The statistical method is also explicitly mentioned in the Materials and Methods section.
