# Supplementary material for: ZIF-8-coated 3D-printed PCL/ion-doped BCP scaffolds for enhanced bone regeneration
Source: PLoS One. 2026 Jun 11;21(6):e0347048. doi: 10.1371/journal.pone.0347048 (PMC13257968; doi:10.1371/journal.pone.0347048)
Supplement: Peer review history

# Peer review: Decision Letter 0

**DOI:** 10.1371/journal.pone.0347048.r001

**Authors:** Tapash Rautray

6 Jan 2026

PONE-D-25-64571

ZIF-8-Coated 3D-Printed Ion-Doped BCP/PCL Scaffolds for Enhanced Bone Regeneration

PLOS One

Dear Dr.  Dini,

Thank you for submitting your manuscript to PLOS ONE. After careful consideration, we feel that it has merit but does not fully meet PLOS ONE’s publication criteria as it currently stands. Therefore, we invite you to submit a revised version of the manuscript that addresses the points raised during the review process.

Please submit your revised manuscript by Feb 20 2026 11:59PM. If you will need more time than this to complete your revisions, please reply to this message or contact the journal office at plosone@plos.org. When you're ready to submit your revision, log on to https://www.editorialmanager.com/pone/ and select the 'Submissions Needing Revision' folder to locate your manuscript file.. When you're ready to submit your revision, log on to https://www.editorialmanager.com/pone/ and select the 'Submissions Needing Revision' folder to locate your manuscript file.

Please include the following items when submitting your revised manuscript:

- A letter that responds to each point raised by the academic editor and reviewer(s). You should upload this letter as a separate file labeled 'Response to Reviewers'.
- A marked-up copy of your manuscript that highlights changes made to the original version. You should upload this as a separate file labeled 'Revised Manuscript with Track Changes'.
- An unmarked version of your revised paper without tracked changes. You should upload this as a separate file labeled 'Manuscript'.

If you would like to make changes to your financial disclosure, please include your updated statement in your cover letter. Guidelines for resubmitting your figure files are available below the reviewer comments at the end of this letter.

If applicable, we recommend that you deposit your laboratory protocols in protocols.io to enhance the reproducibility of your results. Protocols.io assigns your protocol its own identifier (DOI) so that it can be cited independently in the future. For instructions see: https://journals.plos.org/plosone/s/submission-guidelines#loc-laboratory-protocols. Additionally, PLOS ONE offers an option for publishing peer-reviewed Lab Protocol articles, which describe protocols hosted on protocols.io. Read more information on sharing protocols at . Additionally, PLOS ONE offers an option for publishing peer-reviewed Lab Protocol articles, which describe protocols hosted on protocols.io. Read more information on sharing protocols at https://plos.org/protocols?utm_medium=editorial-email&utm_source=authorletters&utm_campaign=protocols..

We look forward to receiving your revised manuscript.

Kind regards,

Tapash Ranjan Rautray

Academic Editor

PLOS One

Journal Requirements:

When submitting your revision, we need you to address these additional requirements.

1.Please ensure that your manuscript meets PLOS ONE's style requirements, including those for file naming. The PLOS ONE style templates can be found at

https://journals.plos.org/plosone/s/file?id=wjVg/PLOSOne_formatting_sample_main_body.pdf and

https://journals.plos.org/plosone/s/file?id=ba62/PLOSOne_formatting_sample_title_authors_affiliations.pdf

2. We notice that your supplementary figures are uploaded with the file type 'Figure'. Please amend the file type to 'Supporting Information'. Please ensure that each Supporting Information file has a legend listed in the manuscript after the references list.

3. If the reviewer comments include a recommendation to cite specific previously published works, please review and evaluate these publications to determine whether they are relevant and should be cited. There is no requirement to cite these works unless the editor has indicated otherwise.

Additional Editor Comments:

Based on the  comments of the Reviewers, I suggest the authors to modify their manuscript.

[Note: HTML markup is below. Please do not edit.]

Reviewer's Responses to Questions

**Comments to the Author**

1. Is the manuscript technically sound, and do the data support the conclusions?

The manuscript must describe a technically sound piece of scientific research with data that supports the conclusions. Experiments must have been conducted rigorously, with appropriate controls, replication, and sample sizes. The conclusions must be drawn appropriately based on the data presented.

Reviewer #1: Yes

Reviewer #2: Partly

**********

2. Has the statistical analysis been performed appropriately and rigorously?

Reviewer #1: Yes

Reviewer #2: Yes

**********

3. Have the authors made all data underlying the findings in their manuscript fully available?

The PLOS Data policy requires authors to make all data underlying the findings described in their manuscript fully available without restriction, with rare exception (please refer to the Data Availability Statement in the manuscript PDF file). The data should be provided as part of the manuscript or its supporting information, or deposited to a public repository. For example, in addition to summary statistics, the data points behind means, medians and variance measures should be available. If there are restrictions on publicly sharing data—e.g. participant privacy or use of data from a third party—those must be specified.requires authors to make all data underlying the findings described in their manuscript fully available without restriction, with rare exception (please refer to the Data Availability Statement in the manuscript PDF file). The data should be provided as part of the manuscript or its supporting information, or deposited to a public repository. For example, in addition to summary statistics, the data points behind means, medians and variance measures should be available. If there are restrictions on publicly sharing data—e.g. participant privacy or use of data from a third party—those must be specified.

Reviewer #1: No

Reviewer #2: Yes

**********

4. Is the manuscript presented in an intelligible fashion and written in standard English?

PLOS ONE does not copyedit accepted manuscripts, so the language in submitted articles must be clear, correct, and unambiguous. Any typographical or grammatical errors should be corrected at revision, so please note any specific errors here.

Reviewer #1: Yes

Reviewer #2: Yes

**********

5. Review Comments to the Author

Please use the space provided to explain your answers to the questions above. You may also include additional comments for the author, including concerns about dual publication, research ethics, or publication ethics. (Please upload your review as an attachment if it exceeds 20,000 characters)

Reviewer #1: The authors describe the fabrication of PCL/BCP scaffolds for bone regeneration applications. They investigated the effect of varied Sr, Mg, Si ion doping concentrations and presence of functional ZIF-8 coatings on material mechanical properties and bioactivity. The manuscript is clearly written, the methodology is well described, and the conclusions appear supported by the results.

1. For mechanical testing, what was the orientation of 3D printed sample with respect to the compression testing?

2. For cell viability tests (Section 3.4.1, Figure 6)

a. What was the control sample? The manuscript simply states a non-scaffold sample. Tissue culture plastic? If so, how does the porosity (available surface area for cells to attach) of the control compare to scaffold samples and does this consideration impact your analysis?

b. For Fig. 6, the convention is generally to draw bars between pairs to denote statistical significance in pair-wise tests, and the lack of bars indicates no statistically significant difference. In the text, authors make statements about statistical significance between Scaffolds 1 and 2. Please confirm the accuracy of the analysis in the manuscript or add bars to Fig 6 if appropriate.

3. Section 3.4.2 and Figure 7 present fluorescence imaging of cells on scaffolds with and without ZIF-8. The authors claim “A higher density of nuclei was observed on scaffold 2 (Fig. 7c) compared to scaffold 1 (Fig. 7a), indicating enhanced cytocompatibility” and “The scaffold 2 (Fig. 7d) exhibited more extensive and denser action filament distribution compared to the scaffold 1…” It is unclear how these findings were reached based on the data presented in Fig 7. Are these findings based on quantitative image analysis (cell counts? fluorescence intensity)?

4. Was statistical analysis performed in Fig 9? If so, please include the results of that analysis in the Figure.

Reviewer #2: Khademi et al present a bone regeneration scaffold that has been 3D printed from polycaprolactone (PCL) and biphasic calcium phosphate (BCP) doped with Sr2+, Mg2+ and Si4+ cations. The scaffold was subsequently coated with a zeolitic imidazolate framework (ZIF-8) consisting if zinc ion and 2-methylimidazole. The mechanical properties of the scaffolds were investigated and cell culture studies were completed.

Overall, I find the work is worth publication. However, I believe it requires major revision prior to publication as an adequate discussion and literature context of the results obtained in this study are lacking. I recommend the authors address the following comments:

(1) On page 4 in Section 2.2.2, how long was the BCP-PCL mixture typically mixed until the solvent was fully evaporated? At what temperature? With magnetic stirring or shaking? Also how were the authors able to confirm that a "uniform solid composite" was obtained?

(2) On page 5 in Section 2.2.4, for the degradation evaluation, what was the control scaffold? Just PCL? And why was degradation only studied in PBS? Wouldn't at least cell culture medium be more representative or the SBF? I would recommend repeating the assay in one or both.

(3) On page 6 in Section 2.4.1, how was blanking done for the cell viability assay?

(4) On page 7 in Section 3.1, I do not understand the criteria for choosing the 40wt% BCP version. What mechanical properties/profile would be optimal for bone grafting and osteocyte differentiation? Such cell types are sensitive to substrate mechanical properties. What does the literature say, how do these results compare to other scaffolds previously presented or used clinically?

(5) On page 8 in Section 3.2, the authors mention that a "mildly acidic microenvironment" should "enhance osteoblast activity and bone remodeling." What pH range would acceptable for this? Are there references to support this?

(6) On page 8 in Section 3.2, the authors indicate that the "PBS controls without scaffolds showed stable pH throughout the study." I would highly recommend adding this data to supplementary information.

(7) On page 8 in Section 3.2 lines 15-16, the authors mention that the PCL:BCP ratio is critical to degradation rate. Were other BCP levels investigated other than 40% BCP for degradation? In addition, were the mechanical properties of the various BCP level scaffolds evaluated during degradation? This could be included in SI.

(8) On page 9 in Section 3.3, for the immersion assay in SBF, were controls without Mg, Si and Sr cations completed? And in Figure 3 (f) on page 11 the description mentions PCL + 40% BCP "including Ca, P, Mg, Si and Sr". Do the authors mean ZIF-8 - PCL+ 40% BCP?

(9) On page 11 in Section 3.3, lines 12 - 17 would be good information for the introduction.

(10) On page 12 in Section 3.5, lines 7-8 discuss the shape/morphology and sizes of the ZIF-8 nanoparticles, giving sizes below 300 nm for the nanoparticles. And the authors present Figure 5 with a SEM image. I do not find this image sufficient to quote a size below 300 nm for the nanoparticles. The authors should include images showing more detail of the nanoparticles at a higher magnification to clearly demonstrate the morphology and sizes of the particles.

(11) On page 13 in Section 3.4.1, in lines 11 -12 the authors mention that scaffold 2 has a higher cell viability than scaffold 1. There is no direct statistical comparison from the data in Figure 6 for scaffold 1 and 2. Additionally, the cell viabilities indicated for scaffolds 1 and 2 are significantly above 100%, starting at day 3. At day 3 in the degradation and bioactivity studies, there was a pH spike to higher pH and by day 5, the pH is lower than at day 0/1. Viability assay signals can be enhanced by various factors. pH changes can have an impact on cell viability assays. In addition, reagents such as MTT can also impact the results. I would recommend controls as similar pH as the samples with scaffold to rule out pH effect on cell viability.

(12) On page 15 Figure 7, the digital version of the images seem to indicate that the substrate is also absorbing the fluorescent stain. The background fluorescence on both DAPI and Phalloidin appear similar to the cells in the images. In addition, I would strongly recommend adding higher magnification images of the cells to show the cells better and allow the reader to investigate the cell morphology easier. It also might be beneficial to overlap the images with a live/dead imaging assay to confirm which cells are live and staining.

(13) On page 16 Figure 8, it is mentioned that the images are at 2 different magnifications. What were they? How many samples were observe for the presented images? Are they all from one sample per scaffold or multiple? And finally, Alzirin Red S binds to other divalent ions, such as Mg2+, Sr2+, etc. It might be a good idea to cross-check the results with von Kossa's protocol.

(14) On page 17 in Figure 9 was there a statistical significance found between Scaffold 1 and 2?

**********

6. PLOS authors have the option to publish the peer review history of their article (what does this mean?). If published, this will include your full peer review and any attached files.). If published, this will include your full peer review and any attached files.

If you choose “no”, your identity will remain anonymous but your review may still be made public.

**Do you want your identity to be public for this peer review?** For information about this choice, including consent withdrawal, please see our For information about this choice, including consent withdrawal, please see our Privacy Policy..

Reviewer #1: **Yes:** Yow-Ren ChangYow-Ren Chang

Reviewer #2: No

**********

[NOTE: If reviewer comments were submitted as an attachment file, they will be attached to this email and accessible via the submission site. Please log into your account, locate the manuscript record, and check for the action link "View Attachments". If this link does not appear, there are no attachment files.]

To ensure your figures meet our technical requirements, please review our figure guidelines: https://journals.plos.org/plosone/s/figures

You may also use PLOS’s free figure tool, NAAS, to help you prepare publication quality figures: https://journals.plos.org/plosone/s/figures#loc-tools-for-figure-preparation.

NAAS will assess whether your figures meet our technical requirements by comparing each figure against our figure specifications.

# Author comment: Author response to Decision Letter 1

**DOI:** 10.1371/journal.pone.0347048.r002

6 Feb 2026

Please see the attached file.

Attachment Submitted filename: Response_R1.docx

# Peer review: Decision Letter 1

**DOI:** 10.1371/journal.pone.0347048.r003

**Authors:** Tapash Rautray

26 Mar 2026

ZIF-8-Coated 3D-Printed PCL/Ion-Doped BCP Scaffolds for Enhanced Bone Regeneration

PONE-D-25-64571R1

Dear Dr. Dini,

We’re pleased to inform you that your manuscript has been judged scientifically suitable for publication and will be formally accepted for publication once it meets all outstanding technical requirements.

Within one week, you’ll receive an e-mail detailing the required amendments. When these have been addressed, you’ll receive a formal acceptance letter and your manuscript will be scheduled for publication.

An invoice will be generated when your article is formally accepted. Please note, if your institution has a publishing partnership with PLOS and your article meets the relevant criteria, all or part of your publication costs will be covered. Please make sure your user information is up-to-date by logging into Editorial Manager at Editorial Manager® and clicking the ‘Update My Information' link at the top of the page. For questions related to billing, please contact  and clicking the ‘Update My Information' link at the top of the page. For questions related to billing, please contact billing support..

If your institution or institutions have a press office, please notify them about your upcoming paper to help maximize its impact. If they’ll be preparing press materials, please inform our press team as soon as possible -- no later than 48 hours after receiving the formal acceptance. Your manuscript will remain under strict press embargo until 2 pm Eastern Time on the date of publication. For more information, please contact onepress@plos.org.

Kind regards,

Tapash Ranjan Rautray

Academic Editor

PLOS One

Additional Editor Comments (optional):

I thank the authors to comply with the comments of the Reviewers and improve their manuscript.

Reviewers' comments:

Reviewer's Responses to Questions

**Comments to the Author**

1. If the authors have adequately addressed your comments raised in a previous round of review and you feel that this manuscript is now acceptable for publication, you may indicate that here to bypass the “Comments to the Author” section, enter your conflict of interest statement in the “Confidential to Editor” section, and submit your "Accept" recommendation.

Reviewer #1: All comments have been addressed

Reviewer #2: All comments have been addressed

**********

2. Is the manuscript technically sound, and do the data support the conclusions?

The manuscript must describe a technically sound piece of scientific research with data that supports the conclusions. Experiments must have been conducted rigorously, with appropriate controls, replication, and sample sizes. The conclusions must be drawn appropriately based on the data presented.

Reviewer #1: Yes

Reviewer #2: Yes

**********

3. Has the statistical analysis been performed appropriately and rigorously?

Reviewer #1: Yes

Reviewer #2: Yes

**********

4. Have the authors made all data underlying the findings in their manuscript fully available?

The PLOS Data policy requires authors to make all data underlying the findings described in their manuscript fully available without restriction, with rare exception (please refer to the Data Availability Statement in the manuscript PDF file). The data should be provided as part of the manuscript or its supporting information, or deposited to a public repository. For example, in addition to summary statistics, the data points behind means, medians and variance measures should be available. If there are restrictions on publicly sharing data—e.g. participant privacy or use of data from a third party—those must be specified.requires authors to make all data underlying the findings described in their manuscript fully available without restriction, with rare exception (please refer to the Data Availability Statement in the manuscript PDF file). The data should be provided as part of the manuscript or its supporting information, or deposited to a public repository. For example, in addition to summary statistics, the data points behind means, medians and variance measures should be available. If there are restrictions on publicly sharing data—e.g. participant privacy or use of data from a third party—those must be specified.

Reviewer #1: Yes

Reviewer #2: Yes

**********

5. Is the manuscript presented in an intelligible fashion and written in standard English?

PLOS ONE does not copyedit accepted manuscripts, so the language in submitted articles must be clear, correct, and unambiguous. Any typographical or grammatical errors should be corrected at revision, so please note any specific errors here.

Reviewer #1: Yes

Reviewer #2: Yes

**********

6. Review Comments to the Author

Please use the space provided to explain your answers to the questions above. You may also include additional comments for the author, including concerns about dual publication, research ethics, or publication ethics. (Please upload your review as an attachment if it exceeds 20,000 characters)

Reviewer #1: (No Response)

Reviewer #2: The authors have addressed all of my comments in this draft of the manuscript. Thank you. I recommend publication.

**********

7. PLOS authors have the option to publish the peer review history of their article (what does this mean?). If published, this will include your full peer review and any attached files.). If published, this will include your full peer review and any attached files.

If you choose “no”, your identity will remain anonymous but your review may still be made public.

**Do you want your identity to be public for this peer review?** For information about this choice, including consent withdrawal, please see our For information about this choice, including consent withdrawal, please see our Privacy Policy..

Reviewer #1: **Yes:** Yow-Ren ChangYow-Ren Chang

Reviewer #2: No

**********

# Editor report: Acceptance letter

**DOI:** 10.1371/journal.pone.0347048.r004

**Authors:** Tapash Rautray

PONE-D-25-64571R1

PLOS One

Dear Dr. Dini,

I'm pleased to inform you that your manuscript has been deemed suitable for publication in PLOS One. Congratulations! Your manuscript is now being handed over to our production team.

At this stage, our production department will prepare your paper for publication. This includes ensuring the following:

* All references, tables, and figures are properly cited

* All relevant supporting information is included in the manuscript submission,

* There are no issues that prevent the paper from being properly typeset

You will receive further instructions from the production team, including instructions on how to review your proof when it is ready. Please keep in mind that we are working through a large volume of accepted articles, so please give us a few days to review your paper and let you know the next and final steps.

Lastly, if your institution or institutions have a press office, please let them know about your upcoming paper now to help maximize its impact. If they'll be preparing press materials, please inform our press team within the next 48 hours. Your manuscript will remain under strict press embargo until 2 pm Eastern Time on the date of publication. For more information, please contact onepress@plos.org.

You will receive an invoice from PLOS for your publication fee after your manuscript has reached the completed accept phase. If you receive an email requesting payment before acceptance or for any other service, this may be a phishing scheme. Learn how to identify phishing emails and protect your accounts at https://explore.plos.org/phishing.

If we can help with anything else, please email us at customercare@plos.org.

Thank you for submitting your work to PLOS ONE and supporting open access.

Kind regards,

PLOS ONE Editorial Office Staff

on behalf of

Dr. Tapash Ranjan Rautray

Academic Editor

PLOS One
